# Porous Alpha-Tricalcium Phosphate with Immobilized Basic Fibroblast Growth Factor Enhances Bone Regeneration in a Canine Mandibular Bone Defect Model

**DOI:** 10.3390/ma9100853

**Published:** 2016-10-19

**Authors:** Nobuhiro Kobayashi, Yoshiya Hashimoto, Akihisa Otaka, Tetsuji Yamaoka, Shosuke Morita

**Affiliations:** 1 First Department of Oral and Maxillofacial Surgery, Osaka Dental University, Hirakata 5731121, Japan; n_kobayashi_3009@yahoo.co.jp (N.K.); morita-s@cc.osaka-dent.ac.jp (S.M.); 2 Department of Biomaterials, Osaka Dental University; Hirakata 5731121, Japan; 3 Department of Biomedical Engineering, National Cerebral and Cardiovascular Center Research Institute, Suita 5658565, Japan; otaka@ncvc.go.jp (A.O.); yamtet@ncvc.go.jp (T.Y.)

**Keywords:** alpha-tricalcium phosphate, basic fibroblast growth factor, bone regeneration

## Abstract

The effect of porous alpha-tricalcium phosphate (α-TCP) with immobilized basic fibroblast growth factor (bFGF) on bone regeneration was evaluated in a canine mandibular bone defect model. Identical bone defects were made in the canine mandible; six defects in each animal were filled with porous α-TCP with bFGF bound via heparin (bFGF group), whereas the other was filled with unmodified porous α-TCP (control group). Micro-computed tomography and histological evaluation were performed two, four and eight weeks after implantation. The bone mineral density of the bFGF group was higher than that of the control group at each time point (*p* < 0.05), and the bone mineral content of the bFGF group was higher than that of the control group at four and eight weeks (*p* < 0.05). Histological evaluation two weeks after implantation revealed that the porous α-TCP had degraded and bone had formed on the surface of α-TCP particles in the bFGF group. At eight weeks, continuous cortical bone with a Haversian structure covered the top of bone defects in the bFGF group. These findings demonstrate that porous α-TCP with immobilized bFGF can promote bone regeneration.

## 1. Introduction

Bone defects attributed to severe periodontitis, trauma, and injury are frequently encountered in the fields of oral implant and orthopedics [1,2]. Although autogenous bone grafting is still considered the gold standard for treatment, it has several disadvantages, including the requirement of a second surgery at the donor site and the limited supply of bone [3,4]. Artificial bone grafts are promising alternatives to autogenous bone grafts.

Tricalcium phosphate (TCP) is a bioresorbable bone substitute used for oral implants and in orthopedics. Alpha (α-)TCP is thermodynamically stable at temperatures above 1100 °C and shows higher solubility than β-TCP [5]. Porous TCP also has potential in applications as a space-making material and drug delivery system in bone defects [5]. However, osteoinduction for bone-regeneration materials requires a relatively long time [6,7].

Human recombinant bone morphogenetic protein 2 belongs to the transforming growth factor-beta (TGF-β) superfamily of proteins [8]. It is a key molecule in bone metabolism owing to its ability to induce differentiation of mesenchymal cells into osteoblasts [8]. TGF-β ligands are involved in tumor suppression or progression [9]; a previous report indicated that recombinant human bone morphogenetic protein-2 is also associated with cancer [10]. Basic fibroblast growth factor (bFGF) regulates bone formation and remodeling [11] but is highly unstable in physiological environments; as such, its activity is rapidly reduced after implantation into the body. We recently showed that specific binding of bFGF to heparin and subsequent tissue integration was accelerated by immobilizing bFGF on porous polymeric materials in a mild and biologically safe reaction [12].

However, bFGF administered as a solution does not always have the expected therapeutic effects in tissue regeneration [2]. Biodegradable devices for the treatment of periodontal bone defects, consisting of a combination of recombinant human platelet-derived growth factor BB (rhPDGF-BB) and β-TCP matrix, are currently either on the market or in development [13]. The first product in this class, GEM 21STM (Luitpold Pharmaceuticals, Shirley, NY, USA), contains β-TCP particles and has been approved by the United States Food and Drug Administration [13]. However, rhPDGF-BB is not chemically adsorbed to the particle surface; it is more likely physically entrapped between and within hydrated particles. Thus, a carrier/delivery system is necessary for the binding and controlled release of growth factors such as bFGF.

In this study, we successfully immobilized bFGF on porous α-TCP via heparin and evaluated the effect of this bFGF carrier on bone regeneration in a canine mandibular bone defect model.

## 2. Results

### 2.1. Analysis of Porous Alpha-Tricalcium Phosphate (α-TCP) Particles with Immobilized Basic Fibroblast Growth Factor (bFGF)

**Figure 1:**
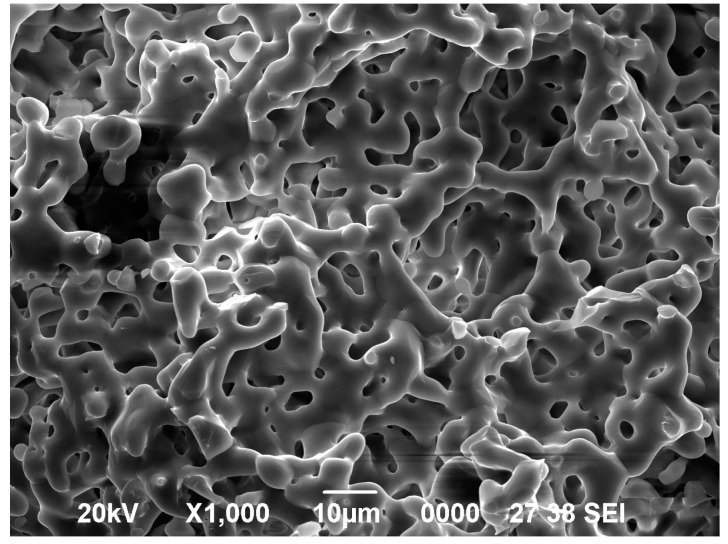
Scanning electron micrographs of alpha-tricalcium phosphate (α-TCP) particles.

**Figure 2:**
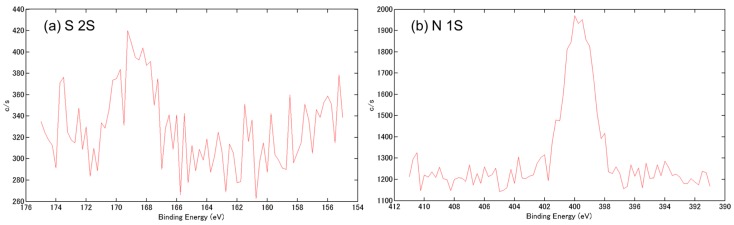
Surface characterization of basic fibroblast growth factor (bFGF) bound to porous α-TCP via heparin. (**a**) X-ray photoelectron spectroscopy (XPS) S 2s and (**b**) XPS N 1s spectra.

The morphology and surface elements of α-TCP before and after the reaction were analyzed by scanning electron microscopy (SEM) and X-ray photoelectron spectroscopy (XPS), respectively. The synthesized α-TCP had a continuous pore structure, with a pore diameter of approximately 5–10 μm, as determined by SEM (Figure 1). A small sulfate peak (S 2s) and a nitrogen peak (N 1s) corresponding to heparin and bFGF, respectively, were detected by XPS analysis of bFGF bound to porous α-TCP via heparin (Figure 2a,b). The amount of immobilized bFGF was quantified using ^125^I-labeled bFGF; we determined that 59.6 ± 3.1 ng of bFGF was immobilized on 1 mg of α-TCP particles.

### 2.2. Three-Dimensional (3D) Microradiography and Bone Mineral Density (BMD) Analysis

**Figure 3:**
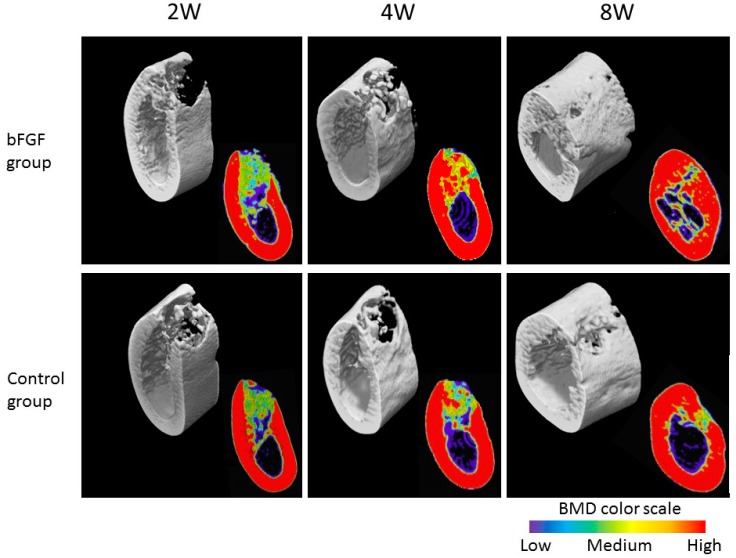
Three-dimensional images and bone mineral density (BMD) analysis of filled defects in the bFGF and control groups at two, four and eight weeks.

A quantitative imageology analysis of newly grown bone was carried out by in vivo 3D microradiography using a bone mineral density (BMD) analysis system. Filled defects in the bFGF and control (unmodified α-TCP) groups at two, four and eight weeks were examined by microradiography (Figure 3). An impermeable structure was observed in both the bFGF and control groups at two and four weeks in the 3D images; the amount of newly formed bone continuously increased so that the entire defect was filled with new bone at eight weeks. The BMD was higher in the bFGF than in the control group at four and eight weeks. In the latter group, the defect was empty, although there was some new bone formation towards the edges of the defect.

**Figure 4:**
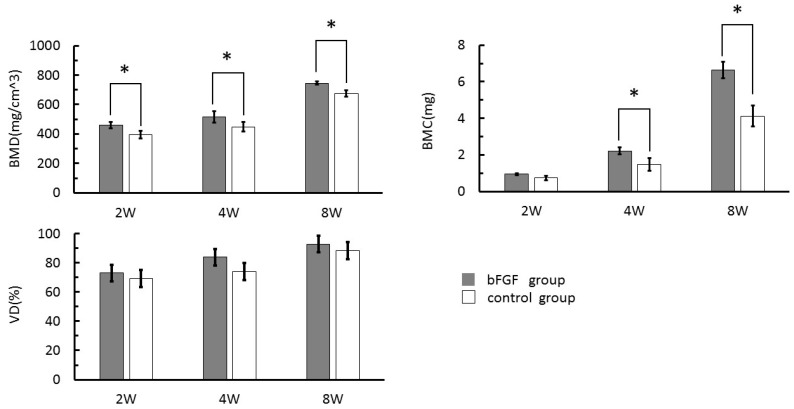
BMD, bone mineral content (BMC), and volumetric density (VD) in the bFGF and control groups at two, four and eight weeks. * *p* < 0.05.

The BMD, bone mineral content (BMC), and volumetric density (VD) of each group were determined at two, four and eight weeks (Figure 4). BMD was higher in the bFGF than in the control group at all time-points examined (*p* < 0.05), whereas BMC was higher in the bFGF group at four and eight weeks (*p* < 0.05) (Figure 4). There were no significant differences in VD between the two groups at any time point (*p* > 0.05) (Figure 4). In addition, the BMD, BMC, and VD were highest at eight weeks in both the bFGF and control groups (*p* < 0.05) (Figure 4).

### 2.3. Histological Assessment

**Figure 5:**
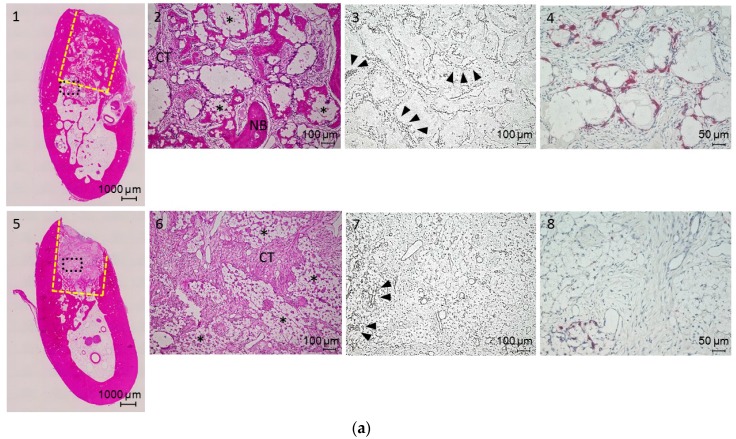

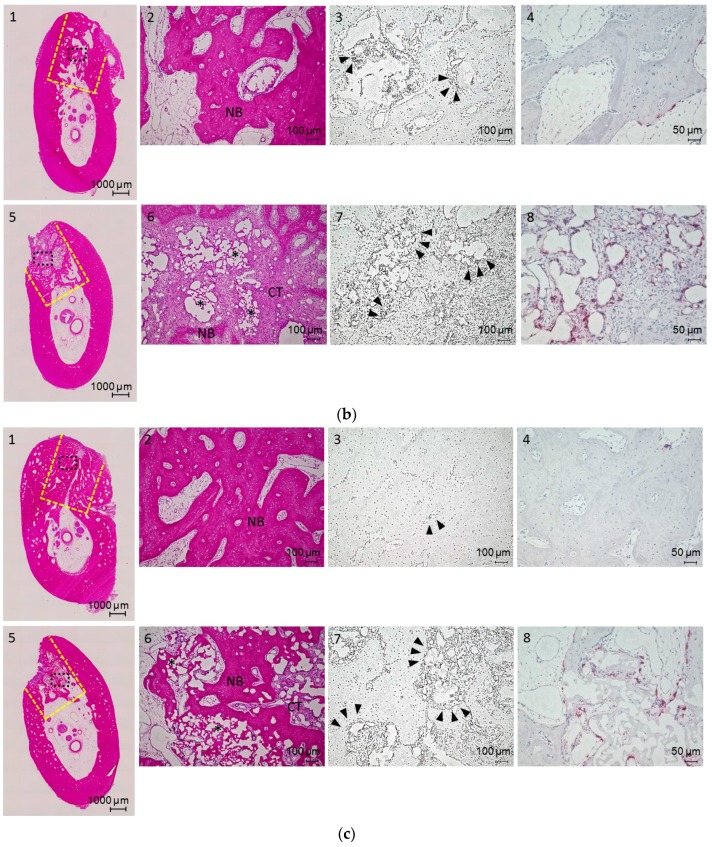
(**a**) Hematoxylin and eosin (H&E)-stained sections (**1**, **2**, **5** and **6**), immunostaining (**3** and **7**), and tartrate-resistant acid phosphatase (TRAP) staining (**4** and **8**) of bone defects in the bFGF (**1**–**4**) and control (**5**–**8**) groups at two weeks; (**b**) H&E-stained sections (**1**, **2**, **5** and **6**), immunostaining (**3** and **7**), and TRAP staining (**4** and **8**) of bone defects in the bFGF (**1**–**4**) and control (**5**–**8**) groups at four weeks; and (**c**) H&E-stained sections (**1**, **2**, **5** and **6**), immunostaining (**3** and **7**), and TRAP staining (**4** and **8**) of bone defects in the bFGF (**1**–**4**) and control (**5**–**8**) groups at eight weeks. CT, connective tissue; NB, newly formed bone; black triangles, new vessels labeled for von Willebrand Factor (vWF); * residual granule. Yellow dotted lines, defect area; black dotted lines, higher magnification area.

**Figure 6:**
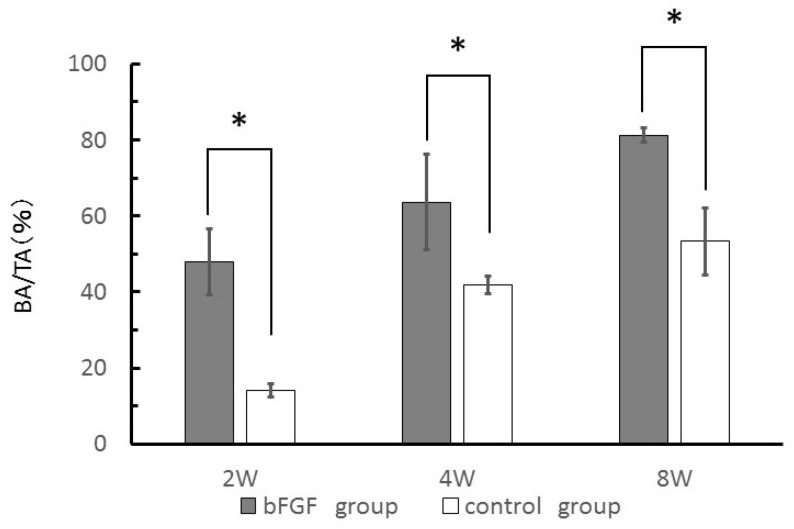
Quantification of new bone growth by measuring new bone area to total area (BA/TA) (%) from H&E-stained sections. * *p* < 0.05.

Newly formed and remodeled bones were subjected to histological analysis. Cross-sections of bone defect sites obtained two, four and eight weeks after surgery were stained with hematoxylin and eosin (H&E) (Figure 5a–c). At two weeks, the presence of bFGF resulted in more bone formation relative to the control group (Figure 5a-1 and -5). In the bFGF group, α-TCP particles were encapsulated with connective tissue and degraded. Newly formed vessels were prominent around the surface of α-TCP particles (Figure 5a-3) and large numbers of osteoclasts showed tartrate-resistant acid phosphatase (TRAP) staining (Figure 5a-4). In the control group, most of the α-TCP particles remained in their original form, and there was no new bone formation (Figure 5a-5 and -6).

**Figure 7:**
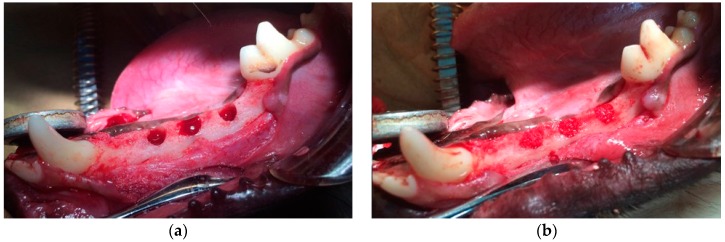
Photographs of (**a**) surgically created and (**b**) treated identical defects.

At four weeks, most of the α-TCP particles in the bFGF group were degraded and replaced with new bone (Figure 5b-1 and -2). New vessels were still observed around residual α-TCP particles (Figure 5b-3 and -4). Few osteoclasts were observed by TRAP staining (Figure 5b-4). In the control group, α-TCP particles were surrounded by connective tissue (Figure 5b-6), which harbored many new vessels (Figure 5b-7). TRAP staining revealed numerous osteoclasts on the α-TCP particle surface (Figure 5b-8).

At eight weeks, new bone with a Haversian structure filled the entire bone defect in the bFGF group (Figure 5c-1) and there were few new vessels (Figure 5c-3). In the control group, α-TCP particles persisted, although some were degraded (Figure 5c-5 and -6), and some new vessels remained (Figure 5c-7). A large number of osteoclasts were detected around the surface of the particles by TRAP staining, and there was some evidence of bone formation (Figure 5c-8).

New bone area (BA) to total area (TA) ratio (BA/TA, %) of each group was measured from H&E-stained sections to quantify new bone growth. The BA of the bFGF group was higher than that of control group at each time point examined (*p* < 0.05) (Figure 6).

## 3. Discussion

bFGF stimulates osteoblast proliferation and differentiation and promotes bone healing at defect sites [11,14,15]. In this study, bFGF was immobilized on porous α-TCP via interaction with heparin. The modified α-TCP was implanted into a canine mandibular bone defect and bone formation and remodeling were evaluated for up to eight weeks. Our previous study [12] showed that bFGF could be immobilized on porous polyethylene specimens via interactions with heparin; here, we confirmed the immobilization of bFGF on α-TCP via heparin by XPS analysis (Supporting Information, Figure 2a,b).

The optimal dose of bFGF is dependent on the carrier that is used. A single injection of 200 μg bFGF was found to promote tibial fracture healing in Beagles [16], and 150 μg bFGF combined with collagen mini-pellets stimulated bone regeneration in dogs [17]. In addition, 100 μg bFGF was the optimal dose for α-TCP implantation in dogs [18]. In this study, mandibular defects were filled with about 70 mg of porous α-TCP particles, corresponding to about 4.2 μg (59.6 ng × 70 mg) bFGF. In our previous study [12], we evaluated bFGF release kinetics from porous polyethylene specimens immobilized with bFGF in a similar manner, and confirmed some bFGF release. We then assessed the stability of bFGF on α-TCP and found that 45% of the bFGF was released from α-TCP in phosphate buffer saline (PBS) in 1 h, while 5% was released over the subsequent seven days.

α-TCP particles have excellent capacity for inducing bone regeneration, but residual material at the site of implantation can either lead to a severe inflammatory response or delay bone healing [19,20,21]. By contrast, rapid dissolution of α-TCP can prevent bone formation [22]. In the bFGF group, new bone formation and vascularization around porous α-TCP was observed by histological examination two weeks after surgery. In our previous study, heparin/bFGF-immobilized porous polycaprolactone scaffolds, with a diameter of 6.0 mm and a thickness of 2.0 mm, were filled with integrated tissue containing many blood vessels just two weeks after implantation into subcutaneous pockets of mice [12]. In another study, bFGF induced the proliferation of osteoblasts and periosteal cells in vitro [23] and stimulated bone formation during the early stages of cranial bone regeneration in a murine model [24]. These findings indicate that bFGF released at the defect site is essential for inducing new bone formation and providing sufficient blood flow to the regenerated bone tissue at the early stage of healing. Thus, porous α-TCP particles with immobilized bFGF maintain an environment that is suitable for osteogenesis and bone regeneration. In this study, TRAP-positive cells—which are involved in bone remodeling—were observed only in the bFGF group two weeks after surgery; these were adjacent to α-TCP particles, indicating that absorption of α-TCP particles leading to bone remodeling was accelerated in the presence of bFGF.

Mandibular defects originate from various pathological processes such as congenital malformations, trauma, oral cancer, and infections. Canines are suitable models for monitoring bone regeneration during correction of mandibular defects, since the sizes and types of deformation that can be created in these animals can approximate those seen in human patients [25]. In addition, bone composition (including ash weight, hydroxyproline, extractable proteins, and insulin-like growth factor-1 content) in dogs is very similar to that in humans [26]. However, bone turnover time in dogs is shorter than in humans, leading to more rapid healing of fractures. Age also influences bone turnover, which is faster in young dogs (such as those used in this study) [27,28]. In our previous study, we created a defect of the same size as the ones in the present study in the canine mandible. In the control group (without implants), there was a small amount of newly woven bone at the edge of the bone defect at two and four weeks, and an appreciable amount of bone formation was also observed at eight weeks [29].

In the present study, the BMC of the bFGF group was 1.5-fold higher than that of controls eight weeks after surgery, whereas bone formation and remodeling were induced in the early stages of recovery (i.e., two weeks after surgery). Our results demonstrate that the release of bFGF from α-TCP particles can effectively reduce the time for bone tissue regeneration. Further studies are required to evaluate the effect of bFGF treatment on periodontal and bone tissue healing, using a clinical model such as two-wall intrabony defects in dogs.

## 4. Materials and Methods

### 4.1. Materials

Porous α-TCP particles with the average diameter of 580.8 μm and porosity of about 80% were obtained from Taihei Chemical Industrial Co. (Osaka, Japan) and sterilized by dry heating before the experiment. The surface-modifier peptide was purchased from SCRUM (Tokyo, Japan). Distilled water and 1000 units/mL heparin solution were supplied by Mochida Pharmaceutical Co. (Tokyo, Japan). bFGF (KCB-1 and Fiblast Spray) were obtained from Kaken Pharmaceutical Co. (Osaka, Japan). Na^125^I solution was purchased from PerkinElmer (Kanagawa, Japan). 

### 4.2. Surface Modification of α-TCP Particles

A predetermined amount of α-TCP particles was added to 0.1% peptide solution in distilled water (1:3 *w*/*v*) and incubated for 24 h at 50 °C in the dark. After rinsing three times with three volumes of distilled water, the particles were dried overnight, and then immersed in heparin solution (1:3 *w*/*v*) for 8 h at room temperature, followed by three rinses as described above and overnight drying in a vacuum. bFGF was immobilized on heparin-modified α-TCP particles by immersion in 1 mg/mL bFGF (Fiblast Spray; Kaken Pharmaceutical Co., Tokyo, Japan) in distilled water (1:3 *w*/*v*) for 24 h at 4 °C followed by three rinses. The procedure was carried out in a sterile environment.

### 4.3. Surface Analysis

Surface morphology and elemental composition of α-TCP particles were evaluated by scanning electron microscopy (JSM-5700; JEOL, Tokyo, Japan) and XPS (ESCA-3400; Shimadzu, Kyoto, Japan), respectively.

### 4.4. Quantification of Immobilized bFGF

The amount of bFGF immobilized on heparin-modified α-TCP particles was quantified by addition of ^125^I-labeled bFGF, which was iodinated by the chloramine-T method, as previously described [12]. Briefly, 12 μL of recombinant human bFGF (KCB-1; 9.95 mg/mL) was mixed with 300 μL chloramine-T in PBS (4 mg/mL) and reacted with 100 μL of Na^125^I solution at a final concentration of approximately 20 MBq/mL. The reaction was terminated with 1 mL sodium persulfate in PBS (10 mg/mL) and purified by ultrafiltration (Amicon Ultra; Millipore, Carrigtwohill, Ireland). The final concentration of ^125^I-labeled bFGF was determined with the bicinchoninic acid assay. The proportion of labeled bFGF was 8.8%. The amount of ^125^I-labeled bFGF immobilized on a given amount of α-TCP was determined based on radioactivity, which was measured with a gamma counter (Cobra Quantum 5003; Packard, Minneapolis, MN, USA).

### 4.5. Canine Mandibular Defect Model

The mandibular defect model was established using six healthy beagles (2 years old, weighing approximately 10 kg) that were obtained from Hamaguchi Animal (Osaka, Japan). The animals were housed in a temperature-controlled environment at 24 °C with free access to food and water. The body weight and general health of the animals were monitored throughout the study.

### 4.6. α-TCP Particle Transplantation

All procedures in this study were approved by the Animal Experiment Committee of Osaka Dental University and conformed to those described in the Guiding Principles for the Use of Laboratory Animals (approval Nos. 12-12001 and 13-03026). The first to fourth premolars were extracted to create space for identical defects 2 months prior to the treatment. An initial incision was made to the squamous epithelium of the gingiva that covered an appropriate part of the mandible, and the subcutaneous tissue was separated from the periosteum. A second incision was made in the periosteum of the mandible, which was lifted and carefully dissected from the underlying mandible. Identical defects (diameter: 4.5 mm, depth: 6 mm) were then made using a twist drill (Astra Tech, Tokyo, Japan) with physiological saline cooled under general anesthesia (0.5 mg/kg pentobarbital sodium) and infiltration anesthesia (1.8 mL of 2% lidocaine hydrochloride and 1:80,000 epinephrine) (Figure 7a). The defects were randomly filled with one of two treatments: bFGF bound to porous α-TCP via heparin, or unmodified porous α-TCP (control) (Figure 7b). The defects were assessed two, four or eight weeks after surgery. The periosteum and skin overlying the defects were sutured in two layers with 3-0 Vicryl (Ethicon GmbH & Co. KG, Norderstedt, Germany) and 3-0 MANI Silk (MANI, Tochigi, Japan). The anti-inflammatory agent Carprofe (CarprodylVR; Ceva, Libourne, France) was administered daily for 7 days following each surgery (tooth extraction and implantation). Six cavities per group were histologically analyzed at each follow-up time point.

### 4.7. Radiographic Analysis

The mandibles were harvested for examination by micro-computed tomography (SMX-130CT; Shimadzu, Kyoto, Japan). Blocks of bone specimen were mounted on the turntable. The exposure parameters were 51 kV and 120 mA. Data obtained from each slice were stored at a resolution of 512 × 512 pixels. TRI/3D BON software (version 7, Ratoc Co., Tokyo, Japan) was used to generate a 3D reconstruction using the volume-rendering method for morphological assessment. In the 3D analysis, the total volume (TV; cm^3^), bone volume (BV; cm^3^), and BMC (mg) were measured using TRI/3D-BON software based on the obtained values. VD was then calculated according to the following formula: VD (%) = BV/TV.

### 4.8. Histological Assessment

The mandibles were fixed in 10% neutral-buffered formalin (Sigma, St. Louis, MO, USA), demineralized in a solution of ethylenediaminetetraacetic acid (Sigma), dehydrated in a graded series of alcohol, and embedded in paraffin. Fixed tissue samples were sectioned (5–7 μm) in the coronal plane and stained with H&E. For immunostaining to detect vascularization, deparaffinized rehydrated sections were treated in 0.1% trypsin and vascular endothelial cells were labeled using a monoclonal anti-human von Willebrand (vW) Factor antibody at a final dilution of 1:1000 (Abcam, Cambridge, UK; ab6994). Immunoreactivity was detected using the EnVision system (Dako, Tokyo, Japan; K4003). Sections were visualized under a BZ9000 All-in-One Fluorescence Microscope (Keyence, Tokyo, Japan). New bone growth was quantified by determining BA/TA (%) in each group based on measurements from images 1 and 5 of Figure 5a–c using ImageJ software (version 1.50i, National Institutes of Health, Bethesda, MD, USA).

### 4.9. Statistical Analysis

Data were evaluated by one-way analysis of variance followed by the Tukey–Kramer post hoc test (OMS Publisher, Tokorozawa, Japan). The Student’s *t*-test was used to compare the values of bFGF and control groups at two, four and eight weeks. *p* < 0.05 was considered significant.
